# Molecular-Level Variation Affects Population Growth in a Butterfly Metapopulation

**DOI:** 10.1371/journal.pbio.0040129

**Published:** 2006-04-25

**Authors:** Ilkka Hanski, Ilik Saccheri

**Affiliations:** **1**Department of Biological and Environmental Sciences, University of Helsinki, Finland; **2**School of Biological Sciences, University of Liverpool, Liverpool, United Kingdom; University of EdinburghUnited Kingdom

## Abstract

The dynamics of natural populations are thought to be dominated by demographic and environmental processes with little influence of intraspecific genetic variation and natural selection, apart from inbreeding depression possibly reducing population growth in small populations. Here we analyse hundreds of well-characterised local populations in a large metapopulation of the Glanville fritillary butterfly *(Melitaea cinxia),* which persists in a balance between stochastic local extinctions and recolonisations in a network of 4,000 discrete habitat patches. We show that the allelic composition of the glycolytic enzyme phosphoglucose isomerase *(Pgi)* has a significant effect on the growth of local populations, consistent with previously reported effects of allelic variation on flight metabolic performance and fecundity in the Glanville fritillary and *Colias* butterflies. The strength and the sign of the molecular effect on population growth are sensitive to the ecological context (the area and spatial connectivity of the habitat patches), which affects genotype-specific gene flow and the influence of migration on the dynamics of local populations. The biological significance of the results for *Pgi* is underscored by lack of any association between population growth and allelic variation at six other loci typed in the same material. In demonstrating, to our knowledge for the first time, that molecular variation in a candidate gene affects population growth, this study challenges the perception that differential performance of individual genotypes, leading to differential fitness, is irrelevant to population dynamics. These results also demonstrate that the spatial configuration of habitat and spatial dynamics of populations contribute to maintenance of *Pgi* polymorphism in this species.

## Abstract 2

Researchers observe a link between the genetic composition of the *Pgi* locus (an enzyme linked with flight metabolic performance) and local population growth within a metapopulation of butterflies.

## Introduction

The notion that a population's genetic composition influences its ecological dynamics is conceptually deeply rooted in population biology [ 1– 3], but to date so few convincing examples have emerged [ 4– 6] that genetic effects on population dynamics are generally thought to be masked or effectively overridden by environmental and demographic processes [ 7]. The exception is inbreeding depression reducing the growth rate of small natural populations, for which there is increasing evidence for both plants [ 8, 9] and animals [ 4, 10] (for a review, see [ 11]). This is not surprising because fitness is generally expected to show an immediate decline with inbreeding, and the demographic consequences may become quickly apparent in small low-density populations. In contrast, the fitness consequences of genetically determined life-history variants are likely to be more dependent on the ecological context and therefore more difficult to detect, and there need not be any demographic consequences at all: natural selection may determine who survives and reproduces, but the number of individuals surviving might be determined by ecological limiting factors (soft selection).

Molecular genetics has started to make an increasing impact in population biology through emphasis on detecting single gene effects in natural populations [ 12] and through application of genomic tools [ 13]. As an example, Osborne et al. [ 14] have reported that two naturally occurring alleles of the *For* gene, which encodes the cGMP-dependent protein kinase, influence foraging behaviour in Drosophila melanogaster larvae and adults. The analogous gene in honeybees influences the regulation of division of labour between forager and nurse bees [ 15]. Fitzpatrick et al. [ 12] discuss other examples in behavioural ecology, but there are no comparable examples of allelic variation in candidate genes influencing population dynamics. A major obstacle to detecting such effects is the need to characterise in some detail the ecological context in which single gene effects or differences in gene expression might have demographic consequences in natural populations.

In this paper we analyse the influence of molecular-level variation on population growth in a large data set from the well-studied metapopulation of the Glanville fritillary butterfly *(Melitaea cinxia)* in the Åland Islands in southwest Finland [ 16]. This metapopulation occurs in a network of approximately 4,000 small habitat patches (dry meadows). The local populations typically consist of less than ten groups of mostly full-sib larvae [ 17], and they have a high risk of extinction. The metapopulation persists in a stochastic balance between extinctions and recolonisations, with around 500 habitat patches being occupied in any given year [ 16, 17]. The processes that influence local dynamics (including local extinction) in this species are well understood and include demographic and environmental stochasticity, habitat loss and alteration, emigration from small habitat patches, parasitism by two specialist parasitoids, and inbreeding depression (for reviews, see [ 16– 18]). Here we use a comprehensive sample representing hundreds of local populations, typed at seven variable genetic loci (five allozymes and two microsatellites), to analyse the possible dependence of annual change in population size on the genetic composition of the respective populations. For reasons explained below, we first focus on the glycolytic enzyme phosphoglucose isomerase *(Pgi)* as a candidate gene but subsequently compare the results for the six other loci typed in the same material.

*Pgi* is an attractive candidate for a polymorphic gene that might influence insect population dynamics. The energetic cost of insect flight is exceptionally high, with some glycolytic enzymes working at rates close to their maximal flux capacity [ 19]. As the catalyst for the interconversion of glucose-6-phosphate and fructose-6-phosphate, *Pgi* plays a key role in glucose metabolism and the resupply of energy (ATP) to flight muscles. A previous study on the Glanville fritillary suggested that the ability to rephosphorylate ADP during active flight is associated with high dispersal rate [ 20]. *Pgi* is often highly polymorphic in natural populations, and it is known to have major effects on fitness in a wide range of taxa [ 21– 24]. Studies on *Colias* butterflies have demonstrated that different allelic combinations (electromorphs) of *Pgi* exhibit distinct kinetic and thermal stability properties [ 25], leading to variation in flight performance [ 26] and associated fitness components [ 27– 29]. In the Glanville fritillary, recent studies have shown that the most frequent *Pgi* genotypes ( *ff, fd, dd*—denoting the homozygotes and heterozygote of the *f* and *d* alleles) differ in their flight metabolic rate and fecundity [ 30].

We show in this paper that the allelic composition of *Pgi,* but not of the other loci studied, has a significant effect on the growth of local populations in the Glanville fritillary, and thus our results challenge the notion that differential performance of individual genotypes, leading to differential fitness, has no consequences for population dynamics. Moreover, the strength and the sign of the molecular effects on population growth are sensitive to the ecological context, the area and spatial connectivity of the habitat patches, which means that detecting such effects requires comprehensive knowledge of the landscape structure and ecology of the study populations. This study exemplifies how molecular genetic assays of candidate genes can be integrated into ecological field studies to address this central question in population biology.

## Results

### Allelic Variation and Population Growth

The design of the genetic sampling was aimed at covering the entire metapopulation and thereby all the ecological situations encountered by the local populations at the time of sampling. The pooled material consists of 1,198 larval families (mostly full-sib), each represented by one larva, sampled from all local populations that were known at the time of sampling ( *n* = 346).

We first describe the results for *Pgi,* then summarise, in the next section, the results for the other loci. The two most common *Pgi* alleles (electromorphs), *d* and *f,* had frequencies of 0.51 and 0.21, respectively, in the pooled material, while five other alleles made up the remaining 0.28. There was much variation among the populations. Considering only the larger populations with more than ten gene copies sampled, the frequency of *f* was <0.06 and >0.42 in the lowest and the highest 10% of populations, respectively.

We describe the *Pgi* composition of local populations by two variables—the sum of the expected frequencies of the *ff* homozygotes and *fd* heterozygotes *,* denoted as *F,* and the expected frequency of the *dd* homozygotes, denoted as *D.* We use *F* to characterise populations because a previous study [ 30] showed that of the three most common genotypes, the *fd* heterozygotes and *ff* homozygotes have a higher flight metabolic rate and are more fecund than the *dd* homozygotes. *F* and *D* ignore genotypes involving the rarer alleles, which have unknown but most likely lower fitness than the *ff* and *fd* individuals [ 30]. Other combinations of genotypes contrasting the presence or absence of the *f* allele produced similar results, which is not surprising because the respective variables are highly correlated (results for the other groupings of genotypes are summarised in the next section).

To have reasonable estimates of *F* and *D* and to minimise the possible impact of inbreeding in the very smallest populations [ 31, 32], we omitted populations with less than six *Pgi* gene copies sampled and hence populations with less than three larval families at the time of sampling. The results were essentially the same in regression models including all of the populations but weighting the regression with the number of gene copies typed ( Table 1, footnote).

**Table 1:**
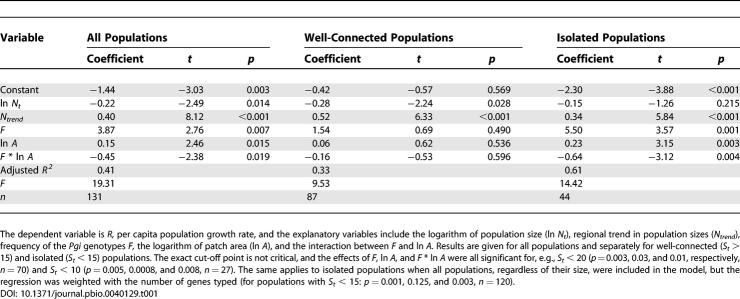
Multiple Regression Models of Population Growth Explained by Ecological Factors and *Pgi* Genotype

Proportional change in population size during one generation was calculated as



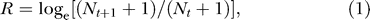



where *N _t_* and *N _t_*_+1_ are the numbers of larval groups in autumns 1995 and 1996, respectively (see Materials and Methods). Of the 131 populations with *N _t_* ≥ 3 (and hence six or more gene copies typed) and for which information about population sizes was available, 34 populations went extinct between the 2 y. Omitting these populations from the analysis would not change the results reported below.

**Figure 1:**
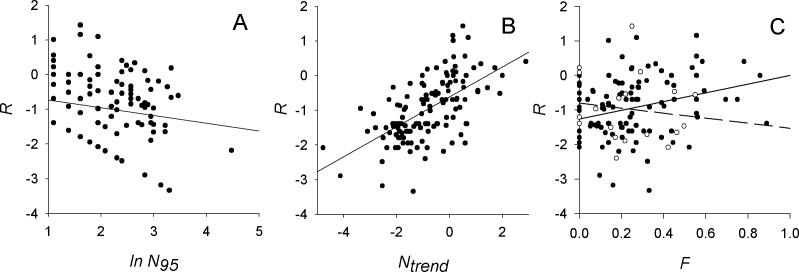
Population Growth Explained by Ecological Factors and *Pgi* Genotype Population growth rate ( *R*) is plotted against the logarithm of population size ( *N _t_*; A), regional change in population sizes ( *N _trend_*; B), and *Pgi* genotype frequency *F* (C). In panel (C), populations in small ( *A* < 0.8 ha) and large habitat patches have been identified with closed (continuous line) and open symbols (broken line), respectively. For statistics, see Table 1.

Considering first the primary ecological factors, the value of *R* was only weakly related to *N _t_* ( Figure 1A) but strongly and positively related to regional change in population sizes in the surroundings of the focal population ( *N _trend_*, definition given in Materials and Methods; Figure 1B). Spatially correlated changes in population sizes are largely due to spatially correlated weather effects [ 17, 33]. Turning then to the effect of allelic variation in *Pgi, R* was positively and significantly related to *F,* and there was a significant interaction between the logarithm of habitat patch area (ln *A*) and *F,* such that *R* increased with *F* in small but decreased with *F* or remained constant in large patches ( Table 1, “All Populations,” and Figure 1C).

### Effect of Allelic Composition in Isolated Populations

In the Glanville fritillary and other comparable butterfly metapopulations, many local populations are so well connected to each other that immigrants make a large contribution to reproduction, often of the order of 50% [ 34, 35]. Therefore, it is only in the more Isolated Populations, receiving only few or no immigrants in a particular generation, that the genotypic composition of populations can be expected to strongly explain the annual change in population size. To test this, we calculated population dynamic connectivity of population *i* as



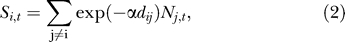



where *N _j,t_* is the number of larval groups in population *j* in autumn 1995, *d _ij_* is the distance between populations *i* and *j,* and 1/ *α* is the average migration distance, set to 1 (km) based on previous studies [ 34, 35]. We divided the populations into those that were isolated ( *S _i,t_* < 15) versus well-connected ( *S _i,t_* > 15) in 1995. The exact cut-off point is not critical ( Table 1, footnote).

**Figure 2:**
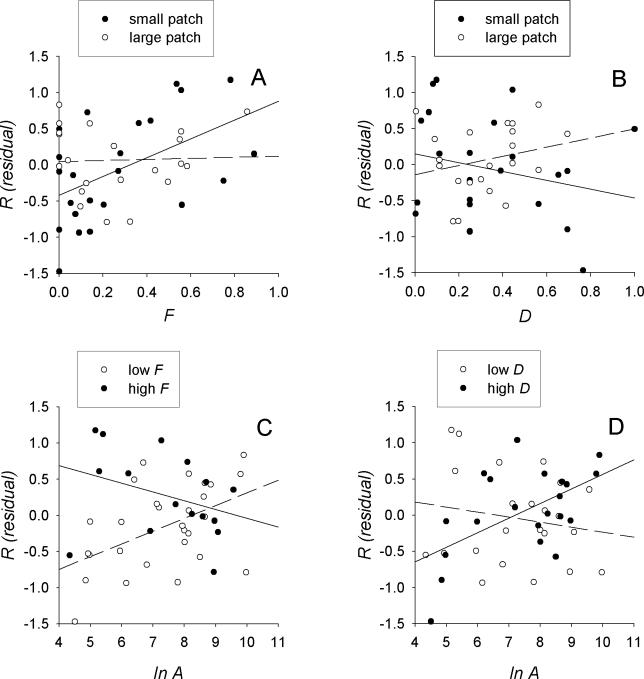
Population Growth Explained by Habitat Patch Area and *Pgi* Genotypes in Isolated Habitat Patches, in which Population Dynamics Are Little Influenced by Immigration The dependent variable in each panel is the residual growth rate ( *R*) from a regression of *R* against regional trend in population sizes ( *N _trend_*), which is explained by genotypic composition and habitat patch area in isolated patches ( *S _t_* < 15). (A and B) show *R* against the *Pgi* genotype frequencies *F* and *D,* respectively, in small ( *A* < 0.3 ha; closed symbols, continuous line) and large habitat patches (open symbols, broken line). (C) shows *R* against patch area in populations with *F* greater (closed symbols, continuous line) or smaller than 0.3 (open symbols, broken line), respectively, and (D) shows the same result for *D.* Statistics on *F* are given in Table 1. In (B and D), the interaction between *D* and ln *A* is significant ( *p =* 0.005; full model *F _3,40_* = 3.64, *p <* 0.02).

A model including *F,* ln *A,* and their interaction explained <1% of variation in *R* in well-connected populations but 17% in isolated populations. In the latter, *R* was highly significantly affected by the F * ln *A* interaction ( Table 1, Figure 2A and 2C: for clarity, the dependent variable in Figure 2 is the residual of *R,* with the dominant effect of *N _trend_* removed). In isolated populations occupying small habitat patches less than 0.2 ha ( *n* = 20), *F* alone explained as much as 48% of variation in *R*. Using the regression model in Table 1 (“Isolated Populations”), we may calculate for representative small habitat patches (0.1 ha), and for the average values of *N _t_* and *N _trend_*, the predicted values of *R* for large (0.9) and small (0.1) values of *F*: 0.85 and 0.36. The ratio of the two values (2.4) gives a rough estimate of the demographic effect of *F* in small patches. The corresponding ratio for representative large patches (0.9 ha) is 0.76; that is, populations in large patches with high *F* perform worse than equivalent populations with low *F* ( Figure 2C). The influence of *D* ( Figure 2B and 2D) was the opposite to that of *F,* which does not follow automatically from the previous results as genotypes other than *ff, fd,* and *dd* account for nearly half of the *Pgi* genotypes.

We repeated the above analysis for the six other loci typed in the same material (see Materials and Methods). The calculations were performed for the frequencies of the two most common alleles at each locus, and for comparison the analysis for *Pgi* was repeated using the frequencies of *f* and *d* instead of *F* and *D*. The result is conclusive: allelic variation in no other locus apart from *Pgi* explained variation in population growth ( Table 2; detailed results given in Table S1).

**Table 2:**
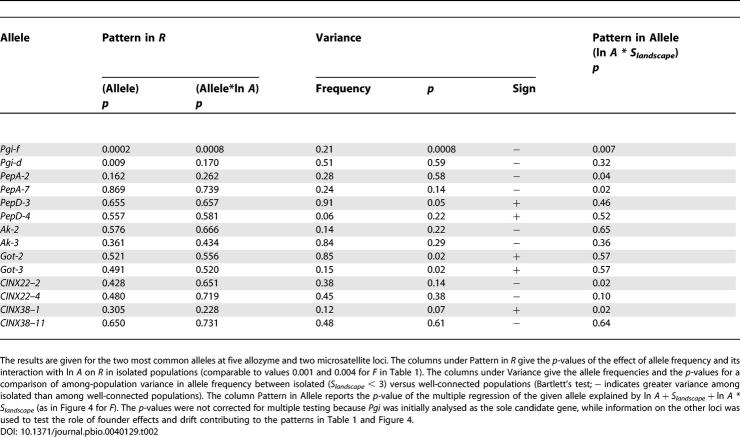
Contrasting Patterns for *Pgi* versus Other Loci, in Relation to Population Growth Rate and the Habitat Structure

Finally, as it is not obvious which combinations of *Pgi* genotypes would best describe the populations for the present purpose, we analysed the effects of alternative groupings of populations' *Pgi* genotypes on their growth using the material for the isolated populations ( *S _i,t_* < 15). Because the frequency of the *f* allele in the metapopulation is relatively low (0.21), many combinations of genotypes involving *f* are highly correlated. For instance, the correlation coefficient between *F* and the frequency of *f* is 0.92 (in the set of 139 populations with *N _t_* ≥ 3). Although we do not expect that the present observational data would suffice to critically discriminate which particular *f*-including genotypes have the strongest influence on population dynamics, we calculated alternative models in which we explained *R* with the expected frequency of particular genotype(s), ln *A,* and their interaction. The fraction of variance explained ( *R* ^2^) by models with different genotypes were as follows ( *x _f_* is the frequency of *f*): *F* ( *= x _f_ x _f_* + *2 x _f_ x _d_*) 0.17, *x _f_ x _f_* 0.14, *2 x _f_ x _d_* 0.14, *2 x _f_* (1 − *x _f_*) 0.17, and *x _f_ x _f_* + *2 x _f_* (1 − *x _f_*) 0.19. Using just the frequency of *f* gave *R ^2^* = 0.18. Thus several models of various groupings involving the *f* allele explain population growth equally well.

### Influence of Habitat Patch Area on the *Pgi* Effect

The positive effect of *F* on *R* is consistent with the greater average clutch size of *fd* and *ff* individuals than of *dd* individuals [ 30], but why should habitat patch area interact with a population's genotypic composition to influence its dynamics? The patch area effect is not due to a correlated effect of population size, although larger patches tend to have larger populations [ 17] (correlation coefficient 0.21 between ln *N _t_* and ln *A, n* = 132).

**Figure 3:**
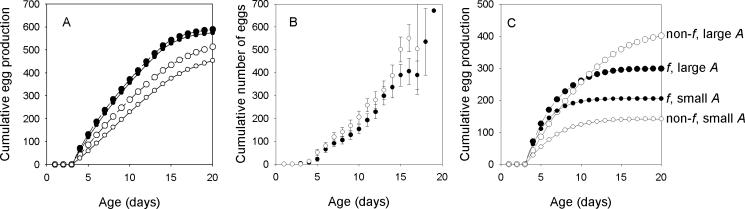
Cumulative Egg Production as a Measure of Reproductive Success of Females with and without the *Pgi-f* Allele (A) Cumulative egg production of *f* (closed symbols) and non- *f* females (open symbols) in small (continuous lines) and large patches (broken lines) in the absence of migration, calculated with the assumptions explained in Materials and Methods. (B) Cumulative egg production of females from new (closed symbols) and old populations (open symbols) in the experiment of Hanski et al. [ 36]. (C) Same as (A) but with the effect of emigration included.

The likely explanation relates to the expected time spent in the natal habitat patch by, and the oviposition schedules of, females with different *Pgi* genotypes, which differ in many life-history traits [ 20, 30, 36]. Using data from a mark-release experiment [ 37] and field cage experiments [ 30, 33], we estimated the values of most of these traits and calculated the expected lifetime egg production of females with and without the *f* allele in small and large habitat patches (see Materials and Methods). The lifetime reproductive outputs thus obtained were 205 and 298 eggs for *f* females and 142 and 401 eggs for non- *f* females, in small and large patches, respectively ( Figure 3). Essentially, the prediction is for *f* females to perform relatively better in small patches due to their faster maturation and higher rate of egg-laying in early life but relatively worse in large patches due to shorter life span and shorter residence time. The interaction between genotype and patch area in egg production is consistent with the comparable interaction between *F* and patch area on population growth ( Figure 2). However, given the large number of life-history traits affected by *Pgi,* it remains a challenge to conclusively prove the actual reasons for the strong genotype–patch area effect.

### Allele Frequency Differentiation in Relation to Patch Area and Connectivity

Females with the *f* allele have a higher flight metabolic rate [ 30] and tend to be more dispersive than females without this allele [ 36]. The former are therefore expected to be particularly common in newly established isolated populations [ 20, 30]. In contrast, isolated populations that have persisted longer than 5 y are known to consist of least dispersive females [ 20], apparently because over the years these populations have lost a large fraction of the more dispersive individuals. On this basis, we would expect more variation in *F* among isolated than well-connected populations. Because the current allele frequencies reflect past dynamics and gene flow rather than just events in the current generation, in this instance we used a measure of landscape connectivity ignoring the occurrence of the butterfly populations in 1995,



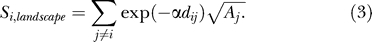



**Figure 4:**
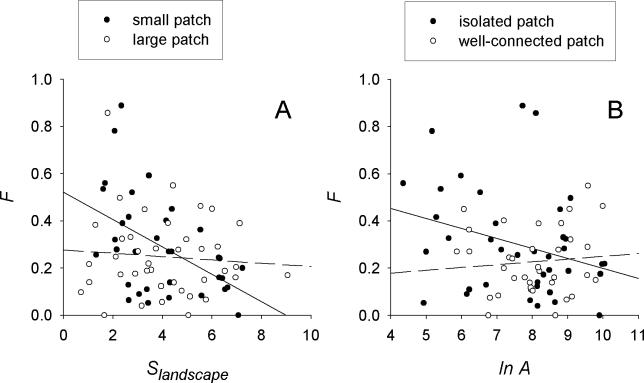
Pattern of *Pgi* Differentiation among Populations The value of *Pgi* genotype frequency *F* in relation to landscape connectivity (A) and habitat patch area (B). Closed (continuous line) and open symbols (broken line) in (A) represent habitat patches smaller or greater than 0.3 ha and in (B) isolated ( *S _landscape_* < 3) and well-connected patches, respectively. A model with ln *A, S _landscape_*, and their interaction explains 14% of variation in *F* ( *F* = 5.00, *p* = 0.003, *n =* 73). The ln A * S _landscape_ interaction ( *t* = 2.54, *p* = 0.01) and the main effects of ln *A* ( *t* = −2.95, *p* = 0.004) and *S _landscape_* ( *t* = −2.84, *p* = 0.006) are all significant. The effects of ln *A, S _landscape_*, and ln A * S _landscape_ were also significant in a model including all populations regardless of their size but weighting the regression with the average population size for the years 1994–1996 ( *F* = 3.24, *p* = 0.02, *n =* 324; the *p-*values for the coefficients were 0.02, 0.01, and 0.03, respectively).

We omitted populations with less than ten gene copies recorded in 1995 to focus on populations that are likely to have persisted for several years (7 y on average, given that the per-year extinction probability of populations with *N _t_* ≥ 5 was 0.15). The results were qualitatively the same in a model with all populations included regardless of their size but the regression weighted with population size ( Figure 4, legend).

As expected, variance among populations in *F* was greater among isolated ( *S _landscape_*< 3) than well-connected populations ( Figure 4A; Bartlett's test, χ ^2^ = 8.27, *df* = 1, *p* = 0.004). Furthermore, *F* increased with isolation in small patches ( Figure 4A) and with decreasing patch area in isolated patches ( Figure 4B). These patterns are consistent with high growth rate of populations with high *F* in small patches and low growth rate in large patches ( Figure 2).

Founder effects and drift could contribute to large variance of *F* among isolated populations, especially in small habitat patches. This was tested by comparing patterns of allele frequency differentiation at *Pgi* with that at the six other, putatively neutral loci, under the assumption that in the absence of selection all loci should be equally affected by drift and migration. Of the 14 most common alleles at the seven loci, only *Pgi-f* shows significantly greater variance among isolated than well-connected populations ( Table 2), which does not support the drift hypothesis, although more generally drift is known to promote genetic structuring in this metapopulation [ 31]. Similarly, the pattern in allele frequency in relation to patch area and connectivity (comparable to Figure 4) is absent in all other loci apart from *Pgi* ( Table 2: ln *A* * *S _landscape_*; more details given in Table S2).

### Inbreeding

Previous studies have shown that inbreeding depression increases the risk of extinction of small local populations of the Glanville fritillary [ 4, 31, 32]. We used the frequency of heterozygous loci *(H _L_*, excluding *Pgi)* in each population as an indirect measure of inbreeding (as in [ 4]). *H _L_* was uncorrelated with *F* ( *r* = 0.03, *n* = 128). Adding *H _L_* to the model in Table 1 (“Isolated Populations”) had a marginally significant ( *p* = 0.05) positive effect on *R* but only if the regression was weighted with population size ( Table S3). We conclude that the effect of *Pgi* on population growth is independent of any inbreeding effect.

## Discussion

The present results demonstrate conclusively that among-population allelic variation at *Pgi,* or variation in linkage disequilibrium with that locus, affects population growth in the Glanville fritillary. There was complete lack of comparable effects in six other loci that were typed in the same material, which underscores the biological significance of the results for *Pgi.* Furthermore, the striking contrast between *Pgi* and the other loci also provides a control for any confounding factors: the present results cannot be explained by population history (founder effects, drift), inbreeding, or the spatial population structure, because all such factors affect all loci in the same manner.

At this stage, we cannot exclude the possibility that the association between allelic variation in *Pgi* and population growth would be due to some other genes linked with *Pgi* rather than *Pgi* itself. For instance, it is possible that *Pgi* is located within an inversion maintaining long-term linkage disequilibrium [ 38]. However, the comparative evidence strongly suggests that *Pgi* is causally involved. Most important, the results on allelic variation in *Pgi* influencing flight metabolic performance, mating success, and oviposition rate are broadly similar for the Glanville fritillary [ 30] and for *Colias* butterflies [ 29], despite these taxa having diverged tens of millions of years ago. If the parallel results were due to loci linked with *Pgi,* the linkage would have to be inconceivably strong. In *Colias,* the different *Pgi* variants show differences in their kinetic properties and thermal stability [ 25] that appear to explain differences in the performance of different genotypes in the field [ 27– 29]. In *Colias eurytheme, Pgi* alleles differ at multiple amino acid sites, but the most consistent differences among electromorphs occur at positions in the loops across the interface between the two monomers of which the molecule consists, which are positions where variation is likely to affect the catalytic properties of the molecule [ 39]. These same sites lie within the only regions of the gene showing strong evidence of selection, based on analysis of nucleotide diversity and hitchhiking by Tajima's D [ 39].

Arguably, even if allelic variation in *Pgi* affects life-history traits as suggested for the Glanville fritillary and *Colias,* it is not necessary that such variation would have consequences for population dynamics. On the other hand, it would also not be very surprising to detect population dynamic consequences of variation in flight metabolic performance and oviposition rate in butterflies. Flight metabolic performance in the Glanville fritillary influences migration rate [ 30, 36], and migration rate influences the dynamics of the mostly small local populations in the large metapopulation that we have studied [ 16]. Density-dependent population regulation is weak in these populations [ 16, 17, 33], which means that variation in fecundity is expected to have an immediate influence on population growth rate.

In addition to demonstrating an effect of molecular variation and natural selection on population dynamics, an important message from this study is context dependence of these molecular effects. First, *Pgi* composition had a strong effect only in isolated populations, in which population dynamics are relatively little affected by migration from other populations. Second, the interaction between allelic composition and habitat patch area is highly significant, apparently reflecting some of the known differences in the performance of different genotypes in small versus large habitat patches. Thus, our results suggest that the population dynamic consequences of populations' *Pgi* composition may be immeasurable in many situations in the field without experimentally perturbing the allelic frequencies, yet the *Pgi* effect may be very apparent and strong in particular situations, such as the numerous small isolated populations in the present study but possibly also in, for example, the expanding front of invasive species.

*Pgi* polymorphisms in butterflies and possibly other taxa may be maintained by heterozygote advantage and a trade-off between enhanced catalytic efficiency and thermal stability among the *Pgi* genotypes [ 29]. Heterozygote advantage and molecular trade-offs may operate also in the Glanville fritillary, but whether they do or not, it is clear that molecular variation in *Pgi* interacts with habitat structure and spatial dynamics to influence population growth, which then again feeds back to influence variation in *Pgi* in the heterogeneous landscape. Thus, this study demonstrates not only that genetic variation and natural selection may influence population dynamics but also that the spatial configuration of habitat and spatial dynamics of populations may contribute to the maintenance of genetic polymorphisms [ 20]. An important challenge for future research is to quantify these statements with metapopulation models explicitly dealing with the *Pgi* locus (and possibly other loci) and parameterised with empirical data of the type in Figure 3. Such a model is needed to fully integrate knowledge of the fitness consequences of molecular variation with spatiotemporal demographic dynamics.

## Materials and Methods

### Larval material for genotyping

In early September 1995, we sampled pre-diapause larvae in their “winter nests” [ 17] by taking one larva from each larval group that was recorded in each local population ( *n* = 346 populations, 1,198 larvae). Roughly 50% of the larval groups are recorded during the annual censuses [ 17]. The larvae were typed for adenylate kinase *(Ak),* glutamate oxaloacetate transferase *(Got),* peptidase A *(PepA),* peptidase B *(PepB),* and *Pgi* using standard methods of cellulose acetate electrophoresis and for two microsatellites (CINX22 and CINX38) [ 31]. We calculated the frequency of heterozygous loci over the four allozymes (excluding *Pgi*) and two microsatellites in each population as a surrogate measure of inbreeding [ 31].

### Population dynamics

Proportional change in population size (measured as the number of larval family groups) was calculated as *R* = log _e_ [( *N _t_*_+ *1*_ + 1)/( *N _t_* + 1)] for populations with at least six copies of *Pgi* typed in the autumn 1995 sample and hence a minimum sample size of three larvae from three larval families (hence minimum *N _t_* was 3). The material included 139 such populations, but we lacked information for eight populations in 1996 and hence these populations had to be omitted. We added “1” to population size to allow the inclusion of populations that went extinct by 1996 ( *n* = 34). Regional change in population sizes in the surroundings of the focal population *i* is defined as *N _i,trend_* = *S _i,t_*_+ *1*_/ *S _i,t_* [ 16], where S _i,*_ is given by equation (2).

### Lifetime reproductive success

We calculated lifetime egg production as a measure of reproductive success of females with and without the *Pgi-f* allele in small and large habitat patches using the following results and assumptions. Among the 139 populations in 1995, the average sizes of “small” and “large” populations were 0.10 and 0.89 ha, respectively, using the limit of *A* = 0.3 ha between the two size classes (as in Figure 2). Below, *f* and non- *f* refer to individuals with and without the *Pgi-f* allele, and *L* and *S* to large and small patches, respectively.

Mating occurs faster in more mobile females [ 36], and many probably less mobile females remain unmated for a long time, especially in small habitat patches with small numbers of males [ 40]. We do not have quantitative data, but the following are plausible values for the daily probability of becoming mated: *f _L_ =* 1.0, *f _S_* = 0.5, non- *f _L_ =* 0.5, non- *f _S_* = 0.25.

In the experiment of Hanski et al. [ 36], the first clutch was laid on day 3 ( Figure 3B). We assume that the daily probability of laying the first clutch (conditional on mating) was 0 for the first 3 d and 0.5 for the subsequent days.

Empirical data collected by M. Saastamoinen (unpublished data) gave the following daily probabilities of laying subsequent clutches, and the average clutch sizes: *f =* 0.4, non- *f =* 0.5, and *f =* 220, non- *f =* 150. These clutch sizes gave higher lifetime egg production than observed in the experiment of Hanski et al. [ 36]; hence we reduced the clutch sizes to 80% of the above values, *f =* 175, non- *f =* 120.

In the experiment described by Hanski et al. [ 36], daily mortality of females from new populations increases sharply after the age of 14 d but much less steeply in females from old populations. The following values estimated by C. Zheng (unpublished data) were used (equating new-population and old-population females with *f* and non- *f* females): *f* females before and after day 14, 0.05 and 0.3; non- *f* females before and after day 14, 0.05 and 0.15.

Butterflies were assumed to stay in the natal patch until they became mated and had laid their first clutch, after which they emigrated with the following daily probabilities: *f _L_ =* 0.2, *f _S_* = 0.4, non- *f _L_ =* 0.05, non- *f _S_* = 0.4. The justifications are as follows. Hanski et al. [ 37] found daily emigration probabilities of 0.05 and 0.11 for females originating from old and new populations from a habitat patch of 0.35 ha. The new populations have a higher frequency of *f* females than old populations [ 30]. We do not have data for the scaling of emigration rate with patch area in *f* and non- *f* females, but it is likely that all butterflies have high emigration rate from very small patches (see [ 41] for a comparison between the sexes in the butterfly Proclossiana eunomia).

These calculations involve many assumptions that merit further study. It is also possible that there are additional behavioural differences among the *f* and non- *f* females. Furthermore, the above calculations ignore the role of males. It is possible that males with the *f* allele are particularly successful mates (as in *Colias* [ 29]), which would further enhance the reproductive success of females in populations with a high frequency of the *f* allele.

## Supporting Information

Table S1 Multiple Regression Models of Population Growth Explained by Ecological Factors and Five Allozymes and Two Microsatellites

Table S2 Patterns of Allelic Differentiation among Populations in Five Allozymes and Two Microsatellites

Table S3 Multiple Regression Model for the Growth of Isolated Populations with the Frequency of Heterozygous Loci Added as a Surrogate Measure of Inbreeding
