# Supplementary material for: Molecular-Level Variation Affects Population Growth in a Butterfly Metapopulation
Source: PLoS Biol. 2006 Apr 25;4(5):e129. doi: 10.1371/journal.pbio.0040129 (PMC1440940; doi:10.1371/journal.pbio.0040129)
Supplement: Supplementary file 1 [file pbio.0040129.st001.doc]

**Supporting information**

# *Population growth explained by allelic variation in 4 other allozymes and 2 microsatellites.* We checked whether the patterns in Figs. 1, 2 and 4 in the main text could be detected in the 4 other autosomal allozymes (*PepA, PepD, Ak,* and *Got*) and in the 2 microsatellites (*CINX22* and *CINX38*) that were scored for this material*.* As explanatory variables, we used the frequencies of the two most common alleles at each locus, and for comparison we repeated the analysis for *Pgi* using the frequencies of *f* and *d* instead of *F* and *D*. Allelic variation in the 4 allozymes excluding *Pgi* and in the 2 microsatellites explained no variation in *R* (Table S1)*.*

# *Allele frequency differentiation in 4 other allozymes and 2 microsatellites.* Table S2 summarizes the results for models explaining the frequency of particular alleles by the logarithm of patch area (ln *A*), landscape connectivity (*S_landscape_*), and their interaction (similar to Fig. 4 in the main text). Only the model involving *Pgi-f* yields a clearly significant result, comparable to that in Fig. 4 for *F*.

***Inbreeding.*** We used the frequency of heterozygous loci (*H_L_*) calculated over the 4 allozymes (excluding *Pgi*) and 2 microsatellites as a surrogate measure of inbreeding. One could expect *H_L_* to have an effect on population growth especially in isolated populations (*S_95_*<15), in which local dynamics are relatively little affected by migration. The effect of *H_L_* was non-significant when added to the model in Table 1 (isolated populations), but it became marginally significant when the multiple regression was weighted by sample size (*N_95_*; Table S3). We conclude that the results on *Pgi* are not due to any effects of inbreeding.

**Table S1.** Multiple regression models of population growth explained by ecological factors and 5 allozymes and 2 microsatellites.

This table reports results similar to those in Table 1 in the main text for the two most common alleles in the 5 allozymes and 2 microsatellites. Multiple regression models for population growth rate (*R*) explained by the logarithm of population size (ln *N_95_*), regional trend in population sizes (*N_trend_*), frequency of the particular allele (frequency in the metapopulation as a whole given in brackets), the logarithm of patch area (ln *A*), and the interaction between allele frequency and ln *A.* Results are given for all populations and separately for isolated (*S_95_*<15) populations.

*Pgi-f* (0.21)

```
		All populations	Isolated
					populations


Coeff    t       P  	Coeff    t       P

Constant 	-1.15	-2.41	 0.017	-2.47	-4.31	<0.001
```

ln *N_95_*	-0.22	-2.48	0.015	-0.19	-1.64	0.110

*N_trend_*	0.40	8.03	<0.001	0.34	6.07	<0.001

```
Pgi-f           3.31 	 1.97 	 0.051 	 7.52 	 4.16 	<0.001
ln A	       	 0.11  	 1.83 	 0.069 	 0.26	 3.66 	 0.001
Pgi-f*ln A     -0.36 	-1.63 	 0.105 	-0.88  	-3.65 	 0.001
```

Adjusted *R^2^*	0.40	0.64

```
F              18.05                 	16.53
n 		  131 			   44
```

*Pgi-d* (0.51)

```
		All populations	Isolated
					populations


Coeff    t       P  	Coeff    t       P

Constant 	-0.32	-0.39	 0.694	 1.88	 1.67	 0.102
```

ln *N_95_*	-0.21	-2.31	0.023	-0.20	-1.38	0.175

*N_trend_*	0.42	8.26	<0.001	0.34	5.49	<0.001

```
Pgi-d         	-0.23 	-0.18 	 0.861 	-4.89 	-2.75 	 0.009
ln A	       	 0.03  	 0.26 	 0.793 	-0.28	-1.88 	 0.067
Pgi-d*ln A      0.01 	 0.08 	 0.938 	 0.64  	 2.50 	 0.017
```

Adjusted *R^2^*	0.37	0.54

```
F              16.20                 	11.07
n 		  131 			   44
```

*PepA-2* (0.28)

```
		All populations	Isolated
					populations


Coeff    t       P  	Coeff    t       P

Constant 	-0.35	-0.66	 0.513	-0.19	-0.26	 0.797
```

ln *N_95_*	-0.19	-2.04	0.043	-0.04	-0.31	0.757

*N_trend_*	0.42	7.93	<0.001	0.40	5.83	<0.001

```
PepA-2       	-0.27 	-0.17 	 0.862 	-2.98 	-1.43 	 0.162
ln A	       	 0.02  	 0.31 	 0.760 	-0.04	-0.41 	 0.682
PepA-2 * ln A   0.02 	 0.10 	 0.922 	 0.33  	 1.14 	 0.262
```

Adjusted *R^2^*	0.34	0.47

```
F              13.99                 	 8.63
n 		  126 			   44
```

*PepA-7* (0.24)

```
		All populations	Isolated
					populations


Coeff    t       P  	Coeff    t       P

Constant 	-0.14	-0.25	 0.800	-0.80	-0.69	 0.497
```

ln *N_95_*	-0.19	-2.07	0.040	-0.10	-0.69	0.495

*N_trend_*	0.42	8.06	<0.001	0.40	5.54	<0.001

```
PepA-7       	-1.16 	-0.75 	 0.455 	-0.42 	-0.17 	 0.869
ln A	       	-0.02  	-0.24 	 0.814 	 0.03	 0.19 	 0.850
PepA-7 * ln A   0.18 	 0.80 	 0.424 	 0.12  	 0.34 	 0.739
```

Adjusted *R^2^*	0.37	0.43

```
F              16.20                 	 7.58
n 		  131 			   44
```

*PepD-3* (0.91)

```
		All populations	Isolated
					populations


Coeff    t       P  	Coeff    t       P

Constant 	-1.57	-0.43	 0.667	 2.64	 0.34	 0.736
```

ln *N_95_*	-0.20	-2.24	0.027	-0.14	-0.94	0.354

*N_trend_*	0.43	8.25	<0.001	0.39	5.20	<0.001

```
PepD-3       	 1.22 	 0.32 	 0.751 	-3.60 	-0.45 	 0.655
ln A	       	 0.13  	 0.27 	 0.784 	-0.37	-0.38 	 0.703
PepD-3 * ln A  -0.10 	-0.20 	 0.838 	 0.45  	 0.45 	 0.657
```

Adjusted *R^2^*	0.35	0.42

*F*	14.99	7.27

*n*	130	44

*PepD-4* (0.06)

```
		All populations	Isolated
					populations


Coeff    t       P  	Coeff    t       P

Constant 	-0.33	-0.81	 0.421	-0.74	-1.17	 0.248
```

ln *N_95_*	-0.21	-2.27	0.025	-0.13	-0.86	0.393

*N_trend_*	0.43	8.22	<0.001	0.39	5.24	<0.001

```
PepD-4       	-1.53 	-0.37 	 0.712 	-5.35 	-0.59 	 0.557
ln A	       	 0.02  	 0.43 	 0.668 	 0.05	 0.65 	 0.517
PepD-4 * ln A   0.17 	 0.33 	 0.745 	 0.64  	 0.56 	 0.581
```

Adjusted *R^2^*	0.35	0.42

```
F              14.87                 	 7.35
n 		  130 			   44
```

*Ak-2* (0.14)

```
		All populations	Isolated
					populations


Coeff    t       P  	Coeff    t       P

Constant 	-0.39	-0.89	 0.374	-1.03	-1.49	 0.143
```

ln *N_95_*	-0.21	-2.36	0.020	-0.15	-1.07	0.292

*N_trend_*	0.43	8.21	<0.001	0.39	5.38	<0.001

```
Ak-2         	 0.23 	 0.09 	 0.927 	 2.02 	 0.56 	 0.576
ln A	       	 0.03  	 0.45 	 0.652 	 0.08	 1.03 	 0.311
Ak-2 * ln A     0.01 	 0.03 	 0.976 	-0.21  	-0.43 	 0.666
```

Adjusted *R^2^*	0.35	0.44

```
F              15.23                 	 7.78
n 		  131 			   45
```

*Ak-3* (0.84)

```
		All populations	Isolated
					populations


Coeff    t       P  	Coeff    t       P

Constant 	 0.45	 0.24	 0.813	 2.07	 0.65	 0.517
```

ln *N_95_*	-0.21	-2.35	0.020	-0.16	-1.13	0.267

*N_trend_*	0.43	8.24	<0.001	0.39	5.34	<0.001

```
Ak-3         	-0.96 	-0.44 	 0.657 	-3.22 	-0.92 	 0.361
ln A	       	-0.06  	-0.23 	 0.817 	-0.27	-0.64 	 0.524
Ak-3 * ln A     0.10 	 0.35 	 0.726 	 0.37  	 0.79 	 0.434
```

Adjusted *R^2^*	0.35	0.44

```
F              15.23                 	 7.99
n 		  131 			   45
```

*Got-2* (0.85)

```
		All populations	Isolated
					populations


Coeff    t       P  	Coeff    t       P

Constant 	-0.69	-0.49	 0.625	-1.99	-1.01	 0.320
```

ln *N_95_*	-0.20	-2.27	0.025	-0.15	-1.02	0.313

*N_trend_*	0.44	8.43	<0.001	0.38	5.16	<0.001

```
Got-2        	 0.41 	 0.26 	 0.799 	 1.51 	 0.65 	 0.521
ln A	       	 0.02  	 0.11 	 0.911 	 0.22	 0.77 	 0.449
Got-2 * ln A    0.01 	 0.01 	 0.996 	-0.20  	-0.59 	 0.556
```

Adjusted *R^2^*	0.36	0.43

```
F              15.79                 	 7.67
n 		  131 			   45
```

*Got-3* (0.15)

```
		All populations	Isolated
					populations


Coeff    t       P  	Coeff    t       P

Constant 	-0.32	-0.76	 0.446	-0.48	-0.61	 0.545
```

ln *N_95_*	-0.20	-2.27	0.025	-0.15	-1.02	0.313

*N_trend_*	0.44	8.37	<0.001	0.38	5.22	<0.001

```
Got-3         	-0.21 	-0.13 	 0.894 	-1.61 	-0.69 	 0.491
ln A	       	 0.03  	 0.51 	 0.614 	 0.02	 0.22 	 0.828
Got-3 * ln A   -0.02 	-0.09 	 0.925 	 0.21  	 0.65 	 0.520
```

Adjusted *R^2^*	0.36	0.43

```
F              15.63                 	 7.69
n 		  131 			   45
```

*CINX22-2* (0.38)

```
		All populations	Isolated
					populations


Coeff    t       P  	Coeff    t       P

Constant 	-1.25	-2.17	 0.032	-1.14	-1.32	 0.195
```

ln *N_95_*	-0.21	-2.31	0.023	-0.12	-0.89	0.380

*N_trend_*	0.44	8.58	<0.001	0.41	5.82	<0.001

```
CINX22-2      	 2.80 	 1.99 	 0.048 	 1.53 	 0.80 	 0.428
ln A	       	 0.14  	 1.81 	 0.072 	 0.07	 0.57 	 0.575
CINX22-2*ln A 	-0.36 	-1.90 	 0.060 	-0.12  	-0.46 	 0.651
```

Adjusted *R^2^*	0.39	0.49

```
F              17.26                 	 8.97
n 		  128 			   43
```

*CINX22-4* (0.45)

```
		All populations	Isolated
					populations


Coeff    t       P  	Coeff    t       P

Constant 	 0.34	 0.47	 0.642	-0.00	-0.00	 0.998
```

ln *N_95_*	-0.21	-2.33	0.022	-0.18	-1.29	0.206

*N_trend_*	0.43	8.26	<0.001	0.41	5.87	<0.001

```
CINX22-4      	-1.55 	-1.12 	 0.265 	-1.59 	-0.71 	 0.480
ln A	       	-0.06  	-0.67 	 0.503 	 0.01	 0.05 	 0.959
CINX22-4*ln A   0.20 	 1.08 	 0.282 	 0.11  	 0.36 	 0.719
```

Adjusted *R^2^*	0.38	0.50

```
F              16.35                 	 9.28
n 		  128 			   43
```

*CINX38-1* (0.12)

```
		All populations	Isolated
					populations


Coeff    t       P  	Coeff    t       P

Constant 	-0.42	-0.92	 0.360	-1.30	-1.89	 0.067
```

ln *N_95_*	-0.21	-2.32	0.022	-0.10	-0.69	0.493

*N_trend_*	0.43	8.51	<0.001	0.38	5.48	<0.001

```
CINX38-1      	 0.11 	 0.04 	 0.966 	 4.40 	 1.04 	 0.305
ln A	       	 0.04  	 0.80 	 0.424 	 0.12	 1.57 	 0.126
CINX38-1*ln A 	-0.10 	-0.32 	 0.752 	-0.71  	-1.23 	 0.228
```

Adjusted *R^2^*	0.38	0.48

```
F              16.61                 	 8.67
n 		  128 			   43
```

*CINX38-11* (0.48)

```
		All populations	Isolated
					populations


Coeff    t       P  	Coeff    t       P

Constant 	-0.99	-1.11	 0.269	-1.16	-1.03	 0.310
```

ln *N_95_*	-0.21	-2.32	0.022	-0.16	-1.05	0.300

*N_trend_*	0.44	8.43	<0.001	0.38	5.43	<0.001

```
CINX38-11     	 1.38 	 0.77 	 0.442 	 1.12 	 0.46 	 0.650
ln A	       	 0.10  	 0.89 	 0.375 	 0.09	 0.60 	 0.553
CINX38-11*ln A -0.17 	-0.72 	 0.470 	-0.11  	-0.35 	 0.731
```

Adjusted *R^2^*	0.37	0.45

```
F              16.14                 	 7.93
n 		  128 			   43
```
