# Supplementary material for: Molecular-Level Variation Affects Population Growth in a Butterfly Metapopulation
Source: PLoS Biol. 2006 Apr 25;4(5):e129. doi: 10.1371/journal.pbio.0040129 (PMC1440940; doi:10.1371/journal.pbio.0040129)
Supplement: Supplementary file 2 [file pbio.0040129.st002.doc]

**Table S2.** Patterns of allelic differentiation among populations in 5 allozymes and 2 microsatellites.

Models explaining the frequency of a particular allele among the populations by the logarithm of patch area (ln *A*), landscape connectivity (*S_landscape_*), and their interaction (similar to Fig. 4 in the main text). Omitting populations with <10 gene copies sampled.

*t*-values

Locus	*F	P*	*n*	ln *A*	*S_landscape_*	interaction

### Pgi-f 4.34 0.007 73 -2.67 -2.46 2.18

```
Pgi-d		1.19	0.32	 73		0.57	 -0.16    0.21
PepA-2	2.93	0.04	 75		0.49	  1.52   -1.13
PepA-7	3.65	0.02	 75	     -2.66	 -2.82    2.58
PepD-3	0.87	0.46	 72	     -0.40	  0.19   -0.25
PepD-4	0.77	0.52	 72		0.72	  0.21   -0.15
Ak-2		0.55	0.65	 75	     -1.09	 -1.24    1.27
Ak-3		1.08	0.36	 75		1.30	  1.45   -1.58
Got-2		0.69	0.57	 75		1.09	  0.52   -0.61
Got-3		0.69	0.57	 75	     -1.09	 -0.52    0.61
CINX22-2	3.63	0.02	 73		2.40    1.39   -1.32
CINX22-4	2.17	0.10	 73		0.65 	  1.30   -1.47
CINX38-1	3.72	0.02	 73		2.35	  1.98   -1.73
CINX38-11	0.57	0.64	 73	     -0.01	 -0.09    0.25
```
