# Supplementary material for: Molecular-Level Variation Affects Population Growth in a Butterfly Metapopulation
Source: PLoS Biol. 2006 Apr 25;4(5):e129. doi: 10.1371/journal.pbio.0040129 (PMC1440940; doi:10.1371/journal.pbio.0040129)
Supplement: Supplementary file 3 [file pbio.0040129.st003.doc]

**Table S3.** Multiple regression model for the growth of isolated populations with the frequency of heterozygous loci added as a surrogate measure of inbreeding.

This model is comparable to that in Table 1 in the main text for isolated populations (*S_95_*<15), but with the frequency of heterozygous loci (*H_L_*) added as a surrogate measure of inbreeding. The regression was weighted by population size in 1995 (*N_95_*), which was excluded as an explanatory variable. *R^2^ =* 0.59, *F* = 13.19, *P*<0.001, *n* = 43.

```
Predictor
variables   	Coeff  	t  	   P


constant       	-2.83     -3.75    0.001
```

*N_trend_*	0.33	5.41	<0.001

```
F                  4.97      2.92    0.006
ln A               0.16      1.96    0.058
F * ln A          -0.56     -2.43    0.020
```

*H_L_*	1.54	2.01	0.052
